# Effectiveness of injecting lower dose subcutaneous sterile water versus saline to relief labor back pain: Randomized controlled trial

**DOI:** 10.18332/ejm/85793

**Published:** 2018-03-14

**Authors:** Howieda Fouly, Ragaa Herdan, Dina Habib, Chao Yeh

**Affiliations:** 1 Obstetrics & Gynecology Nursing, Faculty of Nursing, Assiut University, Egypt; 2 Anesthesia & Intensive Care, Faculty of Medicine, Women’s Health Hospital, Assiut University, Egypt; 3 Obstetrics & Gynecology, Faculty of Medicine, Women’s Health Hospital, Assiut University, Egypt; 4 School of Nursing, Johns Hopkins University, USA

**Keywords:** back pain, labor, subcutaneous injection, effectiveness, lower dose, sterile water

## Abstract

**INTRODUCTION:**

The aim of this study was to investigate the effect of a lower dose subcutaneous sterile water injection technique versus subcutaneous saline injection, on the relief of low-back pain for women during childbirth, and to explore the lasting effects of pain relief after administration (followed at 15, 30, 45, 90 and 120 minutes).

**METHODS:**

A prospective randomized controlled single-blinded study was conducted, with trial registration (NCT02813330). Women received one-time injections (sterile water or saline) and the effectiveness was observed at 15, 30, 45, 90 and 120 minutes after the intervention.

**RESULTS:**

The intervention group had statistically significant pain reduction. Assessment of subsequent pain, followed at 30, 45, 90 and 120 minutes, reflected an increasing change with a statistically significant difference. The intervention group had more burning sensations than the control group with a statistically significant difference.

**CONCLUSIONS:**

The modified technique of double injections of subcutaneous ‘water/ saline’ resulted in significant relief of low-back pain during childbirth.

## INTRODUCTION

Almost more than one-third to 45% of women experience lower-back pain during childbirth^1,2^. This pain, different from pains related to uterine contraction, originates from the lumbosacral area that is supplied by afferent neurons ending in the dorsal horns of spinal subdivisions located at T10-L1 and is a referred pain from the lower back^3^. Lower back pain most likely occurs during latent and early active phases of the first stage of labor^4^.

There has been a significant growth in the use of pharmacological epidural analgesia during childbirth^5^. However, there are disadvantages in using this analgesia, as it has negative influences on maternal and neonatal outcomes^6^ and is not appropriate for all patients, such as women with previous cardiac or respiratory problems^7,8^. Its use by obstetricians may even be limited due to its unavailability, owing to limited resources and high costs^9,10^. In addition, women in labor are often hesitant to use medications to reduce childbirth pain because of concerns about potential harm to the parturient mothers, as the majority of them are affected by unavoidable negative outcomes such as hypotension and non-reassuring fetal heart rates^9,11^. Therefore, effective and safe alternative options are needed to relieve back pain for women during the first stage of labor^12^. Sterile water injection is an alternative option to reduce lower-back pain. It can provide pain relief without negative outcomes. Also, it is suitable and available to use in low resource settings^11^.

The technique of sterile water injection originated in Scandinavian countries to reduce back pain during childbirth, which is rationalized through somatic swelling at the injection site (mechanical irritation on tissues). Patients feel an immediate brief pain sensation for few seconds during the administration of the sterile water injection^13^. This mechanism is interpreted through gate control theory in which pain transmission of nerve impulses from peripheral fibers to the cells of the spinal cord can be restrained. When a less painful stimulus (e.g. subcutaneous injection) is applied into the skin, the larger and faster fibers are stimulated. This stimulus then produces an increase in the activity of these fibers and more receptors cells are enrolled to receive impulses that shut the gate to the smaller nerve fibers and prevent the transmission of information about pain to the central nervous system^3,14^. This would brand subcutaneous sterile water injection as an ultimate pain relief choice for labor in the current procedure of childbirth or in low resource settings.

Several systematic reviews were published by Martensson et al.^15^, who reviewed six trials done on water injection. Also, Fogarty^16^ reviewed the same six studies; Hutton et al.^11^ published a systematic review and meta-analysis for eight trials; Derry et al.^17^, in the recent Cochrane review^17^, analyzed seven clinical trials to ascertain the effectiveness of using SSWI for relieving low-back pain during childbirth. Although all trials confirmed a statistically significant difference between treatment and control groups in pain scores, there is a number of limitations of these trials, such as the small sample size of the participants^11,16-18^. Blind experiment design was not reported in trials^19-21^, which may lead to inaccurate outcomes. Relevant to our study, no trail suggested the use of a different number of injections. In addition, almost all of these studies compared 4 injections versus one, and none of them used two sites for the injections rather than 4 sites. Hence, the current study used a modified number (two) and dose (0.5 mL for each) of subcutaneous water injections to explore the effect of this technique on the relief of lower back pain during labor.

### Hypotheses of the study

**H1:** The lower dose of subcutaneous water injections will achieve significant relief of lower back pain during childbirth.

**H2:** The lower dose of subcutaneous water injections will not achieve a significant relief of lower back pain during childbirth.

### Purpose of study

The study objectives were: 1) To investigate the effect of a lower dose subcutaneous sterile water injection (SSWI) and saline injection technique (SSI) for relief of low-back pain for women during childbirth, and 2) To explore the lasting effects of pain relief after administration (followed at 15, 30, 45, 90 and 120 minutes ).

### Methodology

The randomized control trial was registered at clinicaltrial. gov (NCT02813330). After baseline data were collected, participants received the injection, pregnant women were randomized into the sterile water injection ‘intervention’ or saline water injection ‘control’ group. Outcomes were collected at pre, post, and follow-ups at 15, 30, 45, 90, 120 minutes after the intervention.

### Outcomes of the study

#### Primary outcome

Pain intensity was evaluated with a lower back pain score through a Visual Analogue Scale (VAS), after using a lower dose in only two sites for injection rather than 4 sites reported in previous studies^19,20,22,23^.

#### Secondary outcome

The lasting effects of pain relief were measured after the administration, at 15, 30, 45, 90 and 120 minutes, associated with subcutaneous sterile water injection (SSWI) versus saline procedure.

### Participants

The participants were recruited from pregnant women who were admitted to labor units for childbirth. The eligibility criteria for this study were women who were: 1) aged 18 years or older with spontaneous or induced childbirth at the first stage of labor, 2) Either primipara or multipara with a term singleton pregnancy (between 37 and 41 weeks), 3) suffering from low-back pain with the pain intensity ≥ 6 on a 10-point scale (VAS) during childbirth, and 4) the fetus was in a cephalic presentation.

Exclusion criteria were: 1) multiple pregnancies, 2) malpresentation, 3) previous cesarean section (CS), and 4) thrombocytopenia, which may cause a flow of blood at the injection site.

### Setting

The study was conducted at Woman Health Hospital (WHH), which is the first specialized hospital for women’s health care in Upper Egypt, with a capacity of 300 beds including two labor units (40 beds). One of the two labor units is the emergency labor unit that includes 32 beds in 6 rooms. One room for fetal monitoring, in addition to two labor rooms for normal labor and three operative rooms for cesarean sections. The other unit is the labor ward, which comprised 8 beds and two labor rooms.

### Sample Size

The sample size of 150 participants in each arm was determined to achieve 80% power to detect a clinical significant difference at α=0.05. Scores for pain intensity were on VAS (0-10). The data were derived from a population with (SD) ± 2.5 on VAS, confirmed based on previous metaanalysis^11^. Thus, the sample size considered the dropout, estimated 10% attrition, and therefore we recruited 165 participants in each arm with a total of 330.

## METHODS

This study is based on a lower dose of subcutaneous injections by using two sites for injection rather than four sites, as done in previous studies. Before injection, the base line of pain intensity was evaluated with VAS (Wong-Baker^24^) and documented. After that, the investigator pulled then cleaned the skin with an alcohol wipe. The dose of each injection was 0.5 mL of sterile water in two sites. The two investigators inserted two subcutaneous injections simultaneously, slowly at an angle 45 to 90 degrees to the skin according to the woman’s tissue/fat layers; the first group received 2 × 0.5 mL of sterile water injection.

**Figure 1:**
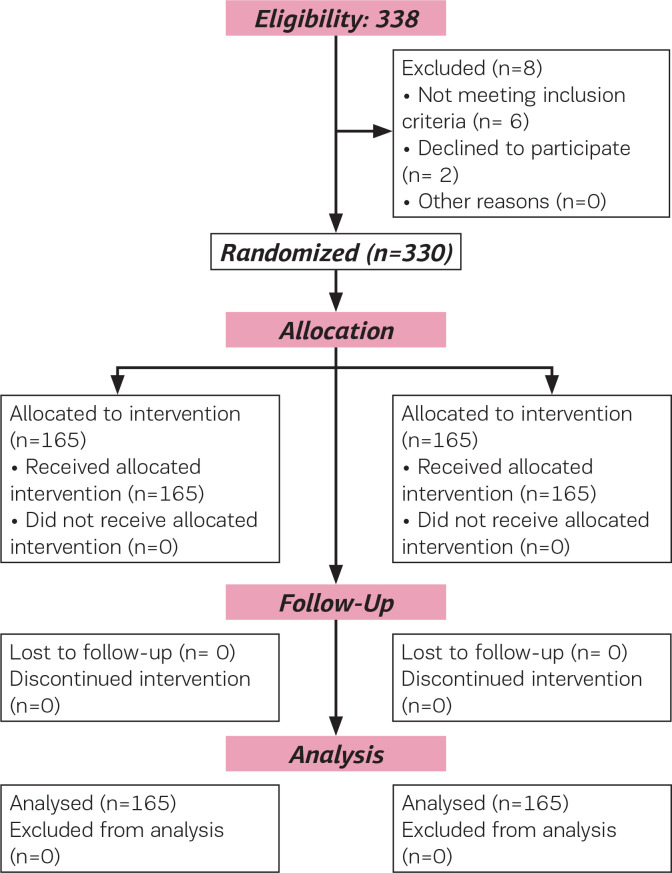
Flow of participants through the trial (CONSORT 2010)

Then two points that extend beyond the area called Michaelis Rhomboid are determined for the injections (Figure 1). Finally, the investigators asked the woman at 15, 30, 45, 90 and 120 minutes, about her pain degree using the scale of pain (VAS). The participants were asked to define degree of pain by choosing a face that described their degree of pain and the investigators registered the pain degree for each participant.

### Sterile water injection ‘intervention’

**Figure 2:**
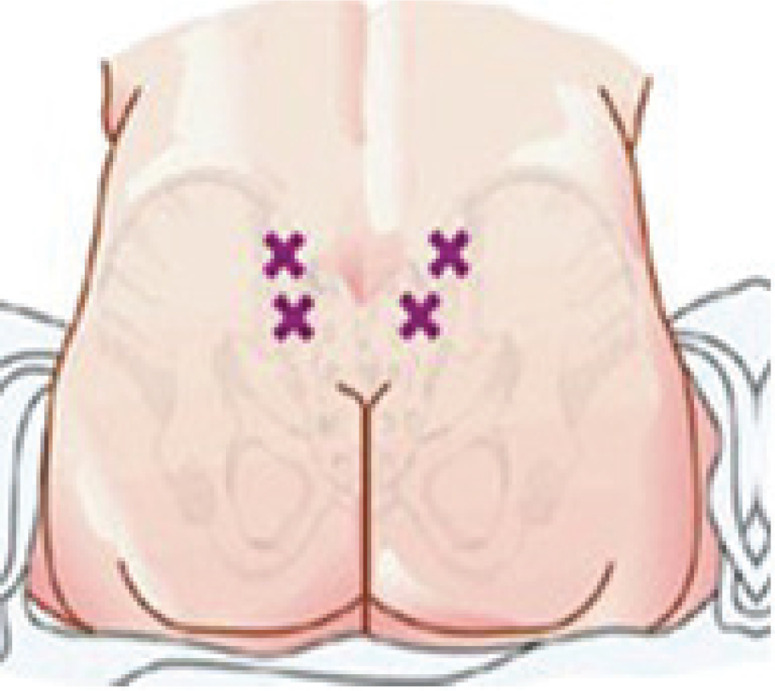
Michaelis Rhomboid points

According to Baxter Healthcare Corporation^25^, a sterile water injection ‘is water for injection sterilized and packaged in single dose vials’. It contains no antimicrobial agents or other preservatives. It is usually used as a diluent. The participants were given two injections of 0.5 mL SWI, simultaneously and subcutaneously, into the area of the Michaelis Rhomboid above the sacral area, after cleaning the skin with an alcohol wipe (Figure 2).

### Saline injection ‘control’

The participants in the control group were given two saline solution injections subcutaneously into the area of Michaelis Rhomboid, above the sacral area. The rationale for choosing saline solution as a placebo was that saline solution has a balanced osmolality of blood ‘isotonic’, which meant it can be distributed rapidly into tissue^26^.

### Technique for injection

Each woman was asked if she preferred to take the injection during contraction or not. The investigators instructed the participant that she might feel a burning sensation during the administration of the injection, which disappears in a few (16-21) seconds but the pain would be relieved within 3-5 minutes and continue up to two hours. Also, women were advised to avoid rubbing the site after injection to avoid fluid leakage^27^.

### Training

The interventionists (HF, PhD nurse; RH, MD physician) were trained through watching videos of subcutaneous injection from previous studies^11,28-30^. Both investigators (HF & RH) had clinical experience of over 15 years in their fields; one is an MD anesthesiologist (RH) and the other is a PhD Obstetrics & Gynecological nursing lecturer (HF). They co-operated during the subcutaneous injection of water/saline.

### Randomization

Study participants were allotted randomly to either one of the two groups via a computer-generated random table. Allocation cover-up was done using an in sequence-numbered sealed opaque envelope. Every envelope was marked with a serial number and contained a card to determine the type of intervention. Once the allocation was done, it could not be changed.

### Blinding

The blinding was followed for both types of injections, and the envelopes of injection procedures were kept in a locker. The investigator could only obtain and open one envelope, then with the assistance of another investigator administered the subcutaneous water/saline injection according to the procedure detailed in the envelope. The participants were not aware of the type of injection, whether it was sterile water or saline water. The nurse who was caring for the woman was asked to be outside the room during injection to ensure the blindness of the procedure.

### Measures

The outcomes were measured by the following tools: 1) Pain Intensity of Visual analogue scale^24^ was used with permission from Wong-Baker Faces Foundation to evaluate the woman’s experience of pain and contained 6 faces marked from 0-10. The respondent was asked to determine the face that represented her pain intensity (0 - no pain, 2 - little pain, 4 - more pain, 6 - lot of pain, 10 - worst pain), and this was repeated after 15, 30, 45, 90 and 120 minutes after injection. Finally, the last part included: outcome of labor such as type of delivery, length of the second stage, third stage, newborn weight, head and chest circumference; 2) Demographics questionnaire: included the patient’s serial number, age, residence, education level, occupation, current obstetric history, such as number of pregnancy, parity, gestational age in weeks, fetal position, cervical dilatation, membrane condition, induction of labor, and assessment of uterine contraction and descend of the head.

### Ethical considerations

The study was approved by the Institutional Review Board, Faculty of Medicine, Assiut University. The consent form explained the nature of the study and benefits related to pain relief after injection. Also mentioned was the tolerable burning sensation during injections, and withdrawal right from the study without risky effects on the woman’s labor process or medication and care received.

### Procedures

After the approval to conduct the study was obtained and the agreement of participants to be included in the study, the baseline data were collected and the participants were randomly assigned to either intervention group or placebo control group. Participants received either sterile water or saline water injections by trained investigators according to group assignment. Study outcomes were collected at every 15, 30, 45, 90 and 120 minutes after the intervention. Study was approved February 2015 and conducted from June to October 2016.

During the injection, the participants were examined for pain sites that were determined by the PhD nurse and the MD anesthesiologist through digital palpation on the painful sites in the lower-back area, mainly over the sacral depressions. Finally, before administration of sterile-water/ saline injection, the participants were asked to express their degree of pain by pointing to the face that described their degree of pain, from a smiling face to a crying face, scaled from 0 to 10, respectively.

Study outcomes were collected at baseline (before the injection), and at 15, 30, 45, 90 and 120 minutes after the injections, thus comprising six data points.

### Study investigator duties

**Figure 3:**
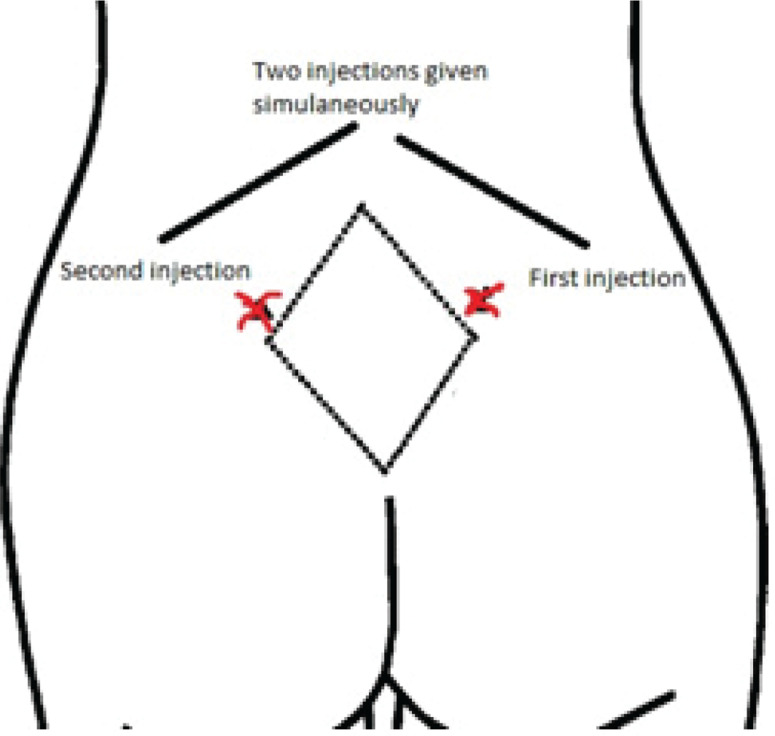
The study sites of water/saline injection

One investigator (HF) prepared the equipment: two syringes (1 mL each) with 25-gauge needles, sterilized water vile, normal saline (NaCl 0.9%) and antiseptic wipes. Then second investigator (RH) placed the woman in a sitting or left lateral position and the other investigator (HF) reassured the woman during the procedure. The sites for injection were determined based on structure displays of the woman’s back. Before the procedure, sacral depressions were palpated for more accuracy. Our study technique used only the lateral two most painful points in the posterior superior iliac crests. An injection was given at the posterior superior iliac crest on the right side and the second injection to the left of the first injection (approximately 2-3 cm apart) (Figure 3).

### Statistical analysis

Statistical analysis was accomplished using SPSS version 20.0 software (IBM Corporation, USA). For comparisons of VAS and pain degree between the two groups, a student t-test was used. Two-way ANOVA test was used to compare pain scores through different times in each group. Fisher’s exact test was used for reported numbers and percentages. Therefore, study categorical data was analyzed via chisquared test. A value of p<0.05 was considered statistically significant. Odds ratio (OR) and 95 % confidence intervals (95 % CI) were calculated. Continuous data were analyzed with a non-parametric test (Mann-Whitney U test).

## RESULTS

### Participant recruitment

A total of 330 participants were contacted for the study. Eight participants were excluded (four for ineligibility and two declined) from participating in the study. Therefore, allocation for the two arms of the study were 165 each. Follow-up and analysis were done for the allocated number in each arm (Figure 1).

### Demographics characteristics of participants

In Table 1 are presented the demographic data of the participants, the subcutaneous sterile water injection (SSWI) group versus subcutaneous saline injection (SSI) group, with mean age of the participants of the two groups 24.6 ± 5.3 and 22.4 ± 4.1 years, respectively. The majority of the two groups lived in rural areas (76% vs 70%). Most participants were housewives (95% vs 89%). The mean education level was 2.9 ± 4.5 vs 7.2 ± 1.2, with a statistically significant difference (p<0.000).

**Table 1:** Demographic characteristics of participants

*Demographic data ‡*	*Intervention Group*	*Control group*	*p*
	*n=165*	*n=165*	
Age (mean ± SD) ^†^	24.6 ± 5.3	22.4 ± 4.1	0.000**
Residence			
Rural n (%)	118 (71.5)	116 (70)	
Urban n (%)	47 ( 28.5)	49 (30)	
Occupation			
House wife	157 (95)	147 (89)	
Employment	8 (5)	18 (11)	
Educational level			
Mean (SD)^‡^	2.9 ± 4.5	7.2 ± 1.2	0.000**
Illiterate (0 years)^€^	38 (22.6)	39 (23.6)	
Read & write (5 years)	14 (8.3)	30 (18.2)	
Primary (9 years)	49 ( 29.2)	58 (35.2)	
Secondary (12 years)	56 (33.3)	37 (22.4)	
University (16 years)	11 (6.5)	1 (0.6)	

‡ SD: Standard Deviation

€ Years: the education level based on numbers of education years.

‡ t-test

** p<0.001

In Table 2 are shown the obstetric data and labor outcomes, between the participant groups. Regarding the gravidity, primigravida represented two-thirds of SSWI (60.6%) and half of SSI (50%) participants, respectively, while the parity in both groups was similar in primiparous (50.3%). In labor outcomes both groups had essentially normal labor, 94.4 and 99.4%, respectively. The weight of the newborns showed that 70.9% for the SSI group and 45.5% for the SSWI weighed more than 3000 g.

**Table 2:** Obstetrics data and labor outcomes between intervention and control groups

*Number of pregnancies (N)*	*Intervention group (SSWI)*		*Control group (SSI)*	
	*N*	*%*	*N*	*%*
Primigravida	100	60.6	83	50.3
More than 1 gravida	65	39.4	82	49.7
Parity				
Primi-paras	83	50.3	83	50.3
Para 1	28	17.0	25	15.2
Multiparas	54	32.7	57	34.5
Labor outcomes				
Normal labor	159	96.4	164	99.4
C.S.	6	3.6	1	0.6
Third stage of labor (duration)				
Less than 20 min	124	75.2	142	86.1
More than 20 min	41	12.4	23	13.9
Newborn weight				
≤ 3000 g	90	54.5	48	29.1
>3000 g	74	45.5	117	70.9

In Table 3 a comparison is given of pain intensity before and after SSWI with time. Participants in the sterile water injection intervention had statistically significant pain reduction (p<0.001), compared to the participants with the saline injection (p<0.001). The subsequent pain assessment after 30, 45, 90 and 120 minutes also showed an increasing absolute change (reaching -40%), with a statistically significant difference (p<0.001).

**Table 3:** Comparison within group effects over time for the two injections

*Pain intensity*	*Water injection*				*Saline Water injection*			
		*Change Score Mean (SD)*	*% change ѣ*	*p*		*Change Score Mean (SD)*	*% change ѣ*	*p*
Baseline	9.35 ± 0.79				9.09 ± 0.93			
VAS 1 (15 min)	7.20 ± 0.91	2.15 ± 1.04	24	0.001**	7.11 ± 0.84	1.97 ± 0.93	22	0.005*
VAS 2 (30 min)	6.43 ± 0.96	2.92 ± 1.10	32	0.001**	6.53 ± 1.12	2.5 ± 1.07	28	0.001**
VAS 3 (45 min)	5.84 ± 0.99	3.48 ± 1.18	37	0.001**	6.20 ± 1.15	2.88 ± 1.4	32	0.001**
VAS 4 (90 min)	5.76 ± 0.70	3.70 ± 1.03	38	0.001**	6.37 ± 7.74	2.85 ± 1.8	31	0.001**
VAS 5 (120min)	6.17 ± 1.02	3.73 ± 1.03	41	0.001**	5. 60 ± 1.13	3.72 ± 1.75	40	0.001**

€ Baseline of pain before subcutaneous sterile water injection.

* p<0.005

** p<0.001

ѣ Difference/change from the baseline of pain score and after injection.

SD: Standard Deviation

Table 4 shows a comparison of pain intensity between intervention (SSWI) and control (SSI) at different times. Using VAS for assessment of pain, a statistically significant difference was obtained between baseline Mean (SD) (9.35 ± 0.73) vs (9.09 ± 0.93) before injection of SWI and SSI, respectively. Pain assessment at 45 and 120 minutes showed a statistically significant difference (p<0.001).

**Table 4:** Comparison between group effects over time for the two injections

	*Intervention*	*Control*	*Mean change^ѣ^*	*p*
	*SSWI Mean*	*SSI Mean*		
Baseline (VAS)^€^	9.35 ± 0.79‡	9.09 ± 0.93	0.26 ± 1.20	0.005*
VAS 1 (15 min)	7.20 ± 0.91	7.11 ± 0.84	0.08 ± 1.20	0.568
VAS 2 (30 min)	6.43 ± 0.96	6.53 ± 1.12	-0.10 ± 1.5	0.405
VAS 3 (45 min)	5.84 ± 0.99	6.20 ± 1.15	-0.35 ± 1.5	0.001**
VAS 4 (90 min)	5.76 ± 0.70	6.37 ± 7.74	-0.81 ± 8.1	0.251
VAS 5 (120 min)	6.17 ± 1.02	5.60 ± 1.13	0.53 ± 1.4	0.001**

€ Baseline of pain before subcutaneous sterile water injection.

* p < 0.005

** p < 0.00

ѣ Difference between the Mean pain measure of each group at labelled times.

‡ SD: Standard Deviation

Table 5 shows the relationship between points of time and pain score after SSWI and SSI. ANOVA test findings showed that the difference in pain score at each point of time from the origin mean score was 2.08, 2.39, 4.43, 6.55 and 2.08, respectively, at 15, 30, 45, 90 and 120 minutes with a statistically significant difference (p<0.000, 0.04, 0.05, 0.20 and 0.00, respectively). Also, saline-test findings showed that the pain score decreased by -5.573, -22.34, -11. 94, -14.21 and -19.22, respectively, at 15, 30, 45, 90 and 120 minutes, with a statistically significant difference (p<0.00, 0.01, 0.00, 0.19 and 0.01, respectively).

**Table 5:** Relationship between points of time and pain score after SSWI and SSI

*Point time of injection*	*Mean square*	*^¥^Difference pain point*	*DF*	*F*	*p*
Before SSWI injection	7.340				
VAS 1 (15 min)	5.252	2.08		6.190	0.00**
VAS 2 (30 min)	4.942	2.39		5.611	0.04*
VAS 3 (45 min)	2.911	4.43	164	3.004	0. 05*
VAS 4 (90 min)		6.55		1.613	0.20
VAS 5 (120 min)				6.190	0.00**
Before SSI injection	7.525					
15 min	13.108	-5.573		23.439	0.00**
30 min	29.866	-22.34		32.415	0.01**
45 min	19.472	-11. 94	162	17.717	0.00**
90 min	97.738	-14.21		1.643	0.197
120 min	26.749	-19.22		34.052	0.01**

* ANOVA test used to measure relationship between points of time injections and pain score * p<0.005

** p<0.00

¥ Difference pain point = Mean square before injection – Mean square after injection = difference of pain at each time point.

**Figure 4:**
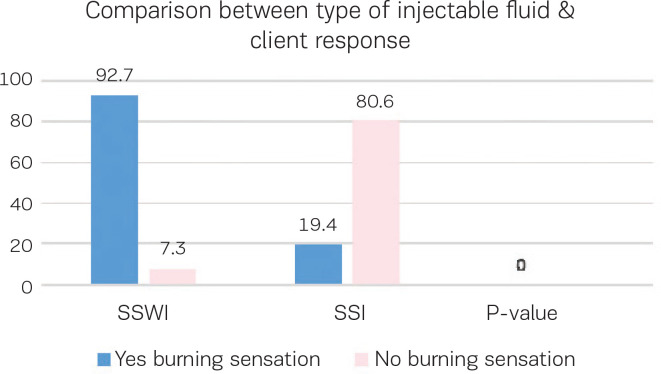
Comparison between type of injectable fluid and client response

Figure 4 illustrates the woman’s feeling of burning sensation during injections of two different fluids water or saline, showing that SSWI has more burning sensation than SSI with a statistically significant difference (p<0.000).

## DISCUSSION

This randomized controlled trial was the first study to use sterile water injections to relieve lower back pain in women using a lower dose through a double injection, rather than four injections as done previously, with women who received saline water injection as the control group. Our findings show that participants in the sterile water injection group reported a gradual decrease of pain intensity after the injection. The change in scores of pain intensity was statistically significantly greater than the scores that participants reported in the control group. The study findings should be interpreted with caution due to the following limitation: we did not compare our new two-sites technique of injection with the four-sites technique used in previous studies. However, our conclusion confirmed statistically significant results related to pain relief using the new two-site injection technique.

The comparison between baseline Means and SDs for the two groups, sterile water injection and saline injection, showed a significant difference, while change in pain percentage achieved an assumed SD (2.4%) in our study that almost matched the assumed SD reported by a metaanalysis study^11^.

The primary outcome of this study was based on the evaluation of pain intensity after injection of SSWI or SSI. Our findings after two hours showed an increasing per cent change, with a statistically significant difference (p<0.000). It was observed that the per cent change with SSWI was better at 15, 30 and 45 min, while SSI was more than SSWI at 90 min after injections. Therefore, this study achieved a good positive result for the use of the lower dose alternatives of ‘sterile water/saline’, for relieving the lower back pain during childbirth.

Our study findings revealed a statistically significant difference between VAS before SSWI and after subsequent assessments (p<0.000). These results match those of Cui et al.^31^, who suggested that sterile water injection has a greater effect on reduction of pain than saline, with a significant difference shown by VAS scores. In addition, it has been reported^29,32-33^ that subcutaneous water injection was shown to provide powerful pain relief, with a statistically significant decrease in pain as early as 10 minutes, and at up to 45 and 90 minutes, following subcutaneous injections of 0.5 mL sterile water. These studies used the four-injections technique.

Our study also used VAS to compare childbirth pain before and after SSI. The comparison shows a statistically significant difference between VAS before SSI and after subsequent assessments. Therefore, these findings are in line with Cui et al.^31^, who showed that normal/isotonic saline injections had a significant effect in relieving lower back pain.

Regarding the adverse effect of using two different types of injectable fluids, either water or saline, on a woman’s feeling of a burning sensation during injection, our results showed that SSWI has a larger burning sensation than SSI, with a statistically significant difference. These findings match studies done by Saxena^28^, who reported that brief pain during injection administration was more noticeable with sterile water than saline. However, the fact that some women asked for more SSWI, despite its burning sensation, shows that injection pain is bearable and does not prevent women from using a sterile water injection. In addition, Cui et al.^31^ reported that the experimental group had a greater change in pre-and post-injections scores than the control group, which revealed a positive experience regardless of pain perception. On the other hand, studies^17,30,34^ have reported that the brief penetrating pain associated with the administration of the sterile water injection had negative effects on participant experiences.

Regarding the effect of the type of injectable fluid on subsequent pain scores, our study showed that both types (water and saline) have a significant effect on pain relief with a difference between both types. These findings are confirmed by similar findings reported^35^ that confirmed that both groups had a reduction in pain scores after injections, comparable to pain before injections, and verified that sterile water had a superior pain-relieving effect compared to that of normal saline.

This study also focused on using two injections which meant a lower dose than for the four sites, reported in previous studies. There were insufficient data to compare our findings related to the number of injections or lower dose of pain relief. However, there was indirect proof found in a study by Martensson et al.^15^ who applied double injection routes, intracutaneous or subcutaneous, to compare them. The indirect proof was related to using two sites for the injections, two for subcutaneous and two for intracutaneous, and their results showed that a woman’s pain was significantly less with subcutaneous injections.

Despite the well documented studies using four sites for sterile water injection versus saline injection to relief back pain during childbirth^19-22,28,29^, we designated two sites in this study. The difference in this study was to use 2 injections rather than 4 injections to explore the effectiveness of a lower dose through less sites to relieve back pain during childbirth. However, the present study did not apply the 4-injections technique to make a comparison with the current technique, since the study relied on the significance of pain score and duration of alternative pain relief, both of which are significant.

## CONCLUSIONS

This study hypothesized that subcutaneous injection of sterile water (at two sites) for relieving low-back pain, throughout childbirth, is better than subcutaneous saline injection. Therefore, the lower dose technique showed a significant relief of a low-back pain throughout childbirth. The only side effect of both types of injection was the tolerable burning sensation during injection. Subcutaneous sterile water injection had more burning sensation than the subcutaneous saline injection, but the effect of pain relief was significantly better than that of the saline injection, supporting the hypothesis of this study. The nurse in this study was at the front line to care for the childbirth women and the nurse was trained to administer SSWI. Therefore, nurses are the ideal health care providers to provide this procedure to women during childbirth. The lower dose technique of two injections needs more research to be applied, especially in developing countries and low resource settings, where women cannot access pain relief during childbirth. This warrants training programs for nurses, midwives and physicians to use this cost-effective method for pain relief during childbirth.

## CONFLICTS OF INTEREST

The authors have completed and submitted the ICMJE Form for Disclosure of Potential Conflicts of Interest and none was reported.
